# Electrical vs Manual Acupuncture Stimulation in a Rat Model of Polycystic Ovary Syndrome: Different Effects on Muscle and Fat Tissue Insulin Signaling

**DOI:** 10.1371/journal.pone.0054357

**Published:** 2013-01-18

**Authors:** Julia Johansson, Louise Mannerås-Holm, Ruijin Shao, AnneLiese Olsson, Malin Lönn, Håkan Billig, Elisabet Stener-Victorin

**Affiliations:** 1 Institute of Neuroscience and Physiology, Department of Physiology, Sahlgrenska Academy, University of Gothenburg, Gothenburg, Sweden; 2 Institute of Biomedicine, Department of Clinical Chemistry and Transfusion Medicine, Sahlgrenska Academy, University of Gothenburg, Gothenburg, Sweden; 3 Department of Obstetrics and Gynecology, First Affiliated Hospital, Heilongjiang University of Chinese Medicine, Harbin, China; VU University Medical Center, The Netherlands

## Abstract

In rats with dihydrotestosterone (DHT)-induced polycystic ovary syndrome (PCOS), repeated low-frequency electrical stimulation of acupuncture needles restores whole-body insulin sensitivity measured by euglycemic hyperinsulinemic clamp. We hypothesized that electrical stimulation causing muscle contractions and manual stimulation causing needle sensation have different effects on insulin sensitivity and related signaling pathways in skeletal muscle and adipose tissue, with electrical stimulation being more effective in DHT-induced PCOS rats. From age 70 days, rats received manual or low-frequency electrical stimulation of needles in abdominal and hind limb muscle five times/wk for 4–5 wks; controls were handled but untreated rats. Low-frequency electrical stimulation modified gene expression (decreased *Tbc1d1* in soleus, increased *Nr4a3* in mesenteric fat) and protein expression (increased pAS160/AS160, Nr4a3 and decreased GLUT4) by western blot and increased GLUT4 expression by immunohistochemistry in soleus muscle; glucose clearance during oral glucose tolerance tests was unaffected. Manual stimulation led to faster glucose clearance and modified mainly gene expression in mesenteric adipose tissue (increased *Nr4a3, Mapk3/Erk, Adcy3, Gsk3b*), but not protein expression to the same extent; however, Nr4a3 was reduced in soleus muscle. The novel finding is that electrical and manual muscle stimulation affect glucose homeostasis in DHT-induced PCOS rats through different mechanisms. Repeated electrical stimulation regulated key functional molecular pathways important for insulin sensitivity in soleus muscle and mesenteric adipose tissue to a larger extent than manual stimulation. Manual stimulation improved whole-body glucose tolerance, an effect not observed after electrical stimulation, but did not affect molecular signaling pathways to the same extent as electrical stimulation. Although more functional signaling pathways related to insulin sensitivity were affected by electrical stimulation, our findings suggest that manual stimulation of acupuncture needles has a greater effect on glucose tolerance. The underlying mechanism of the differential effects of the intermittent manual and the continuous electrical stimulation remains to be elucidated.

## Introduction

Polycystic ovary syndrome (PCOS), a complex female endocrine disorder, is associated with hyperinsulinemia, insulin resistance, dyslipidemia, obesity, and other metabolic derangements [1]. Insulin resistance in PCOS is ascribed to defects in insulin signaling in adipocytes and skeletal muscle [2], [3], [4], [5]. Insulin action through intracellular GLUT4 translocation depends on insulin-dependent pathways involving insulin receptors, insulin receptor substrates, phosphatidylinositol 3-kinase, protein kinase B (Akt), and Akt substrate of 160 kDa (AS160), as well as insulin-independent mechanisms such as muscle contraction/exercise [6], [7]. Insulin-stimulated glucose transport, mediated by glucose transporter 4 (GLUT-4), is reduced in adipocytes of women with PCOS, especially those who are lean [8]. Women with PCOS also have reduced GLUT4 mRNA expression [9] and reduced GLUT4 protein content in whole-cell lysates and membrane preparations of adipose tissue [8], [10].

In clinical studies, low-frequency (2-Hz) electrical stimulation of acupuncture needles placed in skeletal muscles, so-called electro-acupuncture (EA), in combination with manual stimulation of the needles improves endocrine disturbances in women with PCOS [11], [12], [13]. In obese women with PCOS, acupuncture without electrical stimulation is superior to metformin for improving endocrine disturbances, while both treatments improve insulin sensitivity and lipid profile [14]. In experimental studies, muscle contractions elicited by electrical stimulation induce changes in skeletal muscle signaling pathways similar to changes induced by exercise [15], [16], [17], [18]. But when the afferent nerves in the treated hind limb were cut, the increased insulin responsiveness after electrical stimulation was lost, indicating that the response is mediated by activation of afferent nerves rather than by the contractions *per se* [19].

In rats with DHT-induced PCOS, which have insulin resistance, obesity, and a PCOS phenotype including estrous cycle irregularities and ovaries with multiple large atretic antral follicles, low-frequency EA 3 times weekly for 4–5 wks increases insulin sensitivity (euglycemic hyperinsulinemic clamp) and modulates expression of genes related to insulin resistance, obesity and sympathetic activity in adipose tissue [20], [21], [22]. Recently, we demonstrated that intense (5 days/wk) low-frequency EA for 4–5 wks completely restores insulin sensitivity and improves skeletal muscle signaling defects in this model [18]. Moreover, GLUT4 protein expression increased in all compartments of soleus muscle, including the plasma membrane, suggesting an increase in glucose transport capacity. The improved insulin sensitivity after EA in DHT-treated PCOS rats might due in part due to increased expression of GLUT4, which may increase the translocation capability from intracellular compartments to the plasma membrane [18]. Similar results have been demonstrated in Goto-Kakizaki rats and db/db mice, in which low-frequency EA increased insulin sensitivity [16], [23]. Although these initial experimental findings show that electrical stimulation of needles in the muscle can reduce insulin resistance, the underlying signaling mechanism is not identified. Also, it is not known whether manual stimulation of needles in skeletal muscle improves insulin sensitivity to the same extent as electrical stimulation or whether the two types of stimulation have similar effects on regulatory mechanisms in skeletal muscle and adipose tissue.

We hypothesized that repeated acupuncture (5 d per wk for 4–5 wks) with electrical or manual muscle stimulation improves whole-body insulin sensitivity in rats with DHT-induced PCOS, with electrical stimulation being more effective. We also hypothesized that the two stimulation methods have different effects on insulin sensitivity–related signaling pathways in skeletal muscle and visceral adipose tissue. To test these hypotheses, we performed oral glucose tolerance tests (OGTTs), assessed the morphology of pancreatic islets of Langerhans, measured glycogen content in skeletal muscle, adipose tissue, and liver, and analyzed gene and protein expression of molecules related to androgen secretion, glucose transport, MAPK, lipid metabolism, and sympathetic/adrenergic activation in skeletal muscle and adipose tissue.

## Materials and Methods

### Animals, Ethics and Study Procedure

Three Wistar dams, each with 10 female pups (not their biological offspring) were purchased from Charles River (Sulzfeld, Germany). Pups stayed with their lactating dam until 21 d of age and were then housed five per cage under controlled conditions (21–22°C, 55–65% humidity, 12-h light/12-h dark cycle). All rats had free access to tap water and commercial chow (Harlan Teklad Global Diet, 16% protein rodent diet; Harlan Winkelmann, Harlan, Germany). Animal care was based on the principles of the Guide to the Care and Use of Experimental Animals (www.sjv.se). The study was approved by the Animal Ethics Committee of the University of Gothenburg.

At 21 d of age, rats were randomly assigned to three groups: low-frequency EA, manual stimulation, and control (n = 10 per group). Ninety-day continuous-release pellets containing 7.5 mg of DHT (daily dose, 83 µg) (Innovative Research of America, Sarasota, FL) were implanted subcutaneously in the neck to induce PCOS phenotypic characteristics, including metabolic disturbances (insulin resistance and obesity), at adult age [22]. Microchips (AVID, Norco, CA) for numbering and identification were inserted along with the pellets. From 21 d of age throughout the study, body weight was monitored weekly. Acupuncture treatments started at 70 d of age, 7 wks after the start of DHT exposure. The study was concluded after 11–12 wks of DHT exposure, including 4–5 wks of treatment.

### Treatment

Rats were treated daily from Monday to Friday for 4–5 wks (20–25 treatments in total). The duration of treatment was 15 min in week 1, 20 min in wks 2 and 3, and 25 min wks 4 and 5. PCOS rats in the control group were handled in the same way as rats in the electrical and manual muscle stimulation groups except for needle insertion and electrical or manual stimulation. Two acupuncture needles (HEGU Svenska, Landsbro, Sweden) were inserted to a depth of 0.5–0.8 cm in the rectus abdominis and one in each triceps surae muscles bilaterally as described [18], [24], [25]. After insertion, needles in the electrical stimulation group were attached to an electrical stimulator (CEFAR ACU II; Cefar-Compex Scandinavia, Malmo, Sweden) and continuously stimulated at 2 Hz (so-called low-frequency EA) during each treatment session. For manual stimulation, after insertion the needles were rotated back and forth five times every fifth minute during the treatment [26].

### Vaginal Smears

Vaginal smears were obtained daily during the final 2 wks of the experiment, and the stage of cyclicity was determined by microscopic analysis of the predominant cell type [27]. Female rats exposed to DHT are acyclic. During the treatment period, rats were sacrificed in the estrus phase if they showed signs of estrous cycle change.

### Oral Glucose Tolerance Test

Blood glucose was measured at baseline after a 5-h fast (Accu-Chek Compact Plus Glucometer, Roche Diagnostics, Indianapolis, IN). A physiological dose of glucose (0.5 g/ml; total 2 g/kg) (G5146, Sigma-Aldrich, Stockholm, Sweden) was given orally by gavage, and glucose levels were measured 15, 30, 60, and 120 min later. At 0, 15, and 30 min, 10 µl of blood was collected from the tail for ELISA analysis of insulin (90060 Ultra Sensitive Rat Insulin ELISA Kit, Crystal Chem, Downers Grove, IL) and proinsulin (10-1232-01 Rat/Mouse Proinsulin ELISA, Mercodia, Uppsala, Sweden), as recommended by the manufacturer. Glucose tolerance was calculated from the area under the curve, the slope of glucose concentration curve (0–15 min and 15–30 min), and the insulin sensitivity index [28]. Intra- and inter-assay coefficients of variation and sensitivity were (<2%, <2% and 10 mg/dl for glucose, ≤10%, ≤10%, and 0.1 ng/ml for insulin, and 2.5%, 6.3%, and 3 pmol/l for proinsulin.

### Tissue Collection

One hour after the last acupuncture treatment, rats were sedated with isoflurane (2% in a 1∶1 mixture of oxygen and air; Isoba Vet; Schering-Plough, Stockholm, Sweden) and decapitated. The hind limb muscles, soleus, gastrocnemius, and extensor digitorum longus (EDL) were dissected, as were the subcutaneous, inguinal, and visceral fat depots (parametrial, retroperitoneal, and mesenteric), the pancreas, and part of the liver. The tissues were weighed, snap frozen in liquid nitrogen, and stored at –80°C. Portions of the tissue samples were fixed in Histofix containing 6% formaldehyde (Histolab, Göteborg, Sweden) and stored in 70% EtOH.

### Glycogen Content

Glycogen content was measured in skeletal muscle (soleus and EDL) and liver. Snap-frozen tissue was placed in 200 µl of distilled water on ice, homogenized with a pestle, boiled for 5 min, and centrifuged for 5 min at 13,000 rpm. Glycogen levels in the supernatant were analyzed with a colorimetric assay (Glycogen Assay Kit, BioVision, Mountain View, CA).

### Immunohistochemistry of Pancreas

Two paraffin sections, a couple of sections apart, from each animal were placed on Superfrost glass slides, deparaffinized in xylene, rinsed in ethanol, and rehydrated in decreasing concentrations of ethanol. For antigen retrieval, the sections were boiled with an antigen-unmasking solution (Vector Laboratories, Burlingame, CA) for 4 min, heated in a microwave oven for 10 min at 170 W, cooled, rinsed in PBS, and placed in 3% H_2_O_2_ to quench endogenous peroxidase activity. Nonspecific binding was blocked with Background Sniper (Biocare Medical, Concord, CA) for 10 min at room temperature. The sections were incubated in a humidified chamber at room temperature for 1 h with the primary antibody against insulin (A0564 polyclonal guinea-pig; Dako, Glostrup, Denmark; 1∶300). The sections rinsed with PBS and incubated with horseradish peroxidase–conjugated rabbit anti-guinea pig immunoglobulin (P0141, Dako; 1∶50). Immunostaining was visualized with DAB (Vector Laboratories). Sections were counterstained with hematoxylin, dehydrated in increasing concentrations of xylene, and placed on coverslips. Islet area, total section area, and the ratio of islet area to total area were determined with a light microscope (Leica DM 6000B, Leica Microsystems, Wetszlar, Germany) and StereoInvestigator Software V.7 (MBF Bioscience, Williston, VT). The virtual drawing tool was used to manually trace the perimeter of the section/islet, and the area was calculated by the program. Mean values of the two sections were used for statistical analyses.

### RNA Isolation, cDNA Synthesis, and Real-time RT-PCR

Total RNA was extracted from mesenteric adipose tissue and soleus muscle with RNeasy Lipid Tissue Mini Kits and RNeasy Fibrous Tissue Mini Kits (Qiagen, Hilden, Germany) in a QIAcube according to the manufacturer’s protocol. RNA concentrations were determined with a spectrophotometer (ND1000, NanoDrop Technologies, Wilmington, DE). First-strand cDNA in a total volume of 20 µl was synthesized from 1 µg of total RNA using random hexamers (Applied Biosystems, Warrington, UK) and Superscript III reverse transcriptase (Invitrogen Life Technologies, Paisley, UK), all according to the manufacturer’s instructions. To prevent RNase-mediated degradation, RNaseout Recombinant Ribonuclease Inhibitor (Invitrogen) was added to each reaction. For each rat, cDNA reactions were done in triplicate and then pooled to assure good quality. mRNA expression was quantified by real-time RT-PCR and custom TaqMan low-density arrays (Applied Biosystems, Foster City, CA) with primers and probes for 48 selected genes. These genes and the corresponding TaqMan gene expression assay numbers and GenBank accession numbers are listed in Table 1.

**Table 1:** Total selection of putative reference genes, genes related to the androgen and insulin receptor pathway, MAPK activators/inactivators, lipid metabolism, sympathetic/adrenergic pathway and adipokines on the TaqMan low-density arrays including TaqMan gene expression assay number, and GenBank accession number.

Gene Symbol	Gene Description	TaqMan Gene Expression Assay Number	GenBank Accession Number
Putative reference genes			
*Gapdh*	Glyceraldehyde-3-phosphate dehydrogenase	Rn99999916_s1	NM_017008.3
*Ppia*	Peptidylprolyl isomerase A	Rn00690933_m1	NM_017101.1
*Actb*	Beta-actin	Rn00667869_m1	NM_031144.2
*Hprt1*	Hypoanthine guanine phosphoribosyl transferas	Rn01527840_m1	NM_012583.2
Target genes			
Androgen signaling			
*Cyp17a1*	Cytochrome P450, family 17, subfamily a, polypeptide 1	Rn00562601_m1	NM_012753.1
*Cyp19a1*	Cytochrome P450, family 19, subfamily a, polypeptide 1	Rn01422547_m1	NM_017085.2
*Ar*	Androgen receptor	Rn00560747_m1	NM_012502.1
Insulin signaling			
*Insr*	Insulin receptor	Rn00567070_m1	M29014.1
*Irs1*	Insulin receptor substrate 1	Rn02132493_s1	NM_012969.1
*Pik3r1*	Phosphoinositide-3-kinase, regulatory subunit 1 (alpha)	Rn00564547_m1	NM_013005.1
*Pik3cb*	Phosphoinositide-3-kinase, catalytic, beta polypeptide	Rn00585107_m1	NM_053481.1
*Prkaa2*	5-activated protein kinase	Rn00576935_m1	NM_023991.1
*Tbc1d1*	TBC1 domain family, member 1	Rn01413271_m1	XM_341215.4
*Akt2*	Protein kinase B, beta, thymoma viral oncogene homolog 2	Rn00690901_m1	NM_017093.1
*Pdk4*	Pyruvate dehydrogenase kinase isoenzyme 4	Rn00585577_m1	NM_053551.1
*Gsk3b*	Glycogen synthase kinase 3 beta	Rn00583429_m1	NM_032080.1
*Gsk3a*	Glycogen synthase kinase 3 alpha	Rn00569232_m1	NM_017344.1
*Nuak2*	NUAK family, SNF1-like kinase, 2	Rn01759072_m1	NM_001007617.1
*Slc2a4*	Glucose transporter 4	Rn00562597_m1	NM_012751.1
*Mtor*	Mammalian target of rapamycin	Rn00571541_m1	NM_019906.1
*Rps6kb1*	Ribosomal protein S6 kinase, 70kDa, polypeptide 1	Rn00583148_m1	NM_031985.1
*Nr4a1/Nur77*	Orphan nuclear receptor	Rn00577766_m1	NM_024388.1
*Nr4a3*	Orphan nuclear receptor	Rn00581189_m1	NM_031628.1
MAPK activators/inactivators			
*Mapk1/ERK2*	Mitogen-activated protein kinase 1	Rn00671828_m1	NM_053842.1
*Mapk3/ERK1*	Mitogen-activated protein kinase 3	Rn00820922_g1	NM_017347.2
*Mapk14/p38 MAPK*	Mitogen-activated protein kinase 14	Rn00578842_m1	NM_031020.2
*Mapk8/JNK*	Mitogen-activated protein kinase 8	Rn01218952_m1	XM_341399.5
*Dusp1*	Mitogen-activating protein kinase phosphatase 1	Rn00678341_g1	NM_053769.3
*Dusp4*	Mitogen-activated protein kinase phosphatase 4	Rn00573501_m1	NM_022199.1
*Ppargc1a*	Peroxisome proliferator–activated receptor γ coactivator-1 α	Rn01453111_m1	NM_031347.1
*Sirt1*	Sirtuin (silent mating type information regulation 2 homolog) 1 (S. cerevisiae)	Rn01428093_m1	NM_001107627.1
Lipid metabolism			
*Fasn*	Fatty acid synthase	Rn01463550_m1	NM_017332.1
*G6pc*	Glucose-6-phosphatase	Rn00565347_m1	NM_013098.2
*Pparg*	PPAR gamma	Rn00440945_m1	NM_001145366.1/NM_013124.3
*Fabp4*	Adipocyte fatty acid binding protein, aP2	Rn00670361_m1	NM_053365.1
*Lpl*	Lipoprotein lipase	Rn00561482_m1	NM_012598.2
*Lipe*	Lipase, hormone sensitive	Rn00689222_m1	NM_012859.1
Sympathetic/adrenergic pathway			
*Atf2*	Activating transcription factor 2	Rn01276559_m1	NM_031018.1
*Adcy3*	Adenylate cyclase 3	Rn00590729_m1	NM_130779.2
*Adcy4*	Adenylate cyclase 4	Rn00570644_m1	NM_019285.2
*Adrb1*	Adrenergic receptor, beta 1	Rn00824536_s1	NM_012701.1
*Adrb2*	Adrenergic receptor, beta 2	Rn00560650s1	NM_012492.2
*Adrb3*	Adrenergic receptor, beta 3	Rn00565393_m1	NM_013108.1
Adipokines			
*Rbp4*	Retinol binding protein 4	Rn01451318_m1	NM_013162.1
*Adipoq*	adiponectin	Rn00595250_m1	NM_144744.2
*Adipor1*	Adiponectin receptor 1	Rn01483784_m1	NM_207587.1
*Adipor2*	Adiponectin receptor 2	Rn01463177_m1	NM_001037979.1

The low-density arrays were run according to the manufacturer’s protocol. Samples were run in singletons, and each loading port contained 100 ng of total RNA converted to cDNA. The stability of expression of reference genes (*18S*; *Actb*, *Gapdh*, *Hprt1*, *Ppia*) was assessed by using the NormFinder algorithm (http://www.mdl.dk/publicationsnormfinder.htm) to determine the lowest intra- and intergroup variability. The most stable gene combinations were *Gapdh* and *Ppia* (mesenteric adipose tissue) and *Actb* and *Hprt1* (soleus). These combinations served as endogenous controls. Gene expression values were calculated by the 2^–ΔΔ*C*t^ method [29]. The ΔCycle threshold value (ΔCt) was calculated by subtracting the average Ct value of the reference genes from the average Ct value of the target gene. 2^-ΔΔCt^ was estimated as the resulting target gene expression level relative to the expression of the control group. ΔCt values were used for statistical analysis.

### Protein Preparation and Western Blot Analysis

Frozen tissue (soleus muscle and mesenteric adipose tissue) was homogenized with a pellet pestle mixer (Merck, Darmstadt, Germany) in ice-cold buffer (25 mM Tris-HCl, 0.15 M NaCl, 1% Triton-X, 1 mM dithiothreitol, 5 mM EDTA, 0.5 mM phenylmethylsufonyl fluoride, 1% sodium dodecyl sulfate, 200 µM sodium deoxycholate, 10 mM N-ethylmaleimide, 10 mM iodoacetamide) containing 1× complete protease inhibitor cocktail (Roche Diagnostics, Basel, Switzerland). Samples were centrifuged for 30 min at 16.1 x *g* at 4°C, and supernatants were collected. A portion of the supernatant was used for analysis of protein concentration with the BCA protein assay kit (Pierce Biotechnology, Rockford, IL); bovine serum albumin served as the standard. The rest of the supernatant was stored at –80°C.

Aliquots of protein were pretreated with NuPAGE LDS Sample Buffer (Invitrogen, Carlsbad, CA), heated to 70°C for 10 min, and separated on NuPAGE Novex 3–8% Tris-acetate gels (Invitrogen) under reducing conditions with a Tris-acetate buffer system. Proteins were transferred to nitrocellulose membranes (Invitrogen), which were rinsed in Tris-buffered saline containing 0.1% Tween-20 (TBS-T), blocked in 3% albumin fraction V (BSA) (Merck, Darmstadt, Germany) in TBS-T for 1 h at room temperature, and incubated with primary antibody overnight at 4°C. The following antibodies were used: AS160, P-AS160^thr642^, Tbc1D1, horseradish peroxidase–conjugated anti-mouse IgGs (#2670S, #4288S, #4629S, #7076; Cell Signaling Technology, Danvers, MA), GLUT-4 (ab33780, Abcam, Cambridge, UK), Nr4a3 (#pp H7833-00, Perseus Proteomics, Tokyo, Japan), β-actin (A1978, St. Louis, USA), and horseradish peroxidase-conjugated anti-rabbit IgGs (PI-1000, Vector Laboratories, Burlingame, CA). The next day, membranes were rinsed in TBS-T, incubated with the secondary antibodies [α-mouse IgG (#7076, Cell Signaling Technology) or α-rabbit IgG (PI-1000, Vector Laboratories)] for 1 h at room temperature, and rinsed in TBS-T. Protein bands were detected with SuperSignal West Dura Extended Duration Substrate (Pierce Biotechnology) and photographed with the LAS-1000 camera system (Fujifilm, Tokyo, Japan). The intensity of protein signals was quantified by densitometry with MultiGauge software Ver. 3.0; β-actin was used as a loading control and for normalization. Values are expressed in arbitrary densitometric units of relative abundance. Nr4a3 membranes were stripped (30 min, 50°C) in Restore PLUS stripping buffer (Thermo Scientific, Rockford, IL), reblocked with 3% BSA, and reprobed with β-actin.

### Immunofluorescence Staining

The level and location of GLUT4 expression in paraffin-embedded sections of soleus muscle and mesenteric fat were determined by immunofluorescence staining with GLUT4 antibody (ab33780, Abcam) as described [30]. Nuclei were identified by staining with 4′,6-diamidino-2-phenylindole (DAPI). Slides were examined on an Axiovert 200 confocal microscope (Zeiss, Jena, Germany) equipped with the laser-scanning confocal imaging LSM 510 META system (Carl Zeiss) and photomicrographed. Negative control sections were used to adjust background settings. Images of GLUT4 immunoreactivity were adjusted to make optimal use of the dynamic range of detection.

### Statistical Analysis

Values are reported as mean ± SEM or mean ± SD. Body weight gain was analyzed with a mixed between-within subjects ANOVA. The Mann-Whitney U test was used for comparisons of the electrical stimulation, manual stimulation, and control groups. Secondary comparisons were between the two treatment groups (electric vs. manual stimulation). SPSS software (version 17.0, SPSS, Chicago, IL) was used for all statistical analyses. *P*<0.05 was considered significant.

## Results

Four rats, two in the PCOS group and one in each treatment group, were excluded from analysis because they did not have the PCOS phenotype (e.g., had regular cycles, normal ovarian morphology, no obesity, and no insulin resistance). We suspect that the DHT pellets in those rats were faulty.

### Body and Tissue Weight

To assess the effects of manual and electrical stimulation of acupuncture needles on weight gain during treatment in DHT-induced PCOS rats, we used a mixed between-within subjects analysis of variance across the 11-week study period and during 4 wks of treatment. The main effect comparing weight gain in each group was not significant. Body weight at 70 d (before the start of treatment) or at the end point did not differ between the groups (Table 2). The inguinal fat depot weighed less in the electric stimulation group than in the controls (Table 2). In relation to total body weight, the inguinal fat depot weighed less and the soleus muscle weighed more in the low-frequency EA group than in the controls (Table 2).

**Table 2:** Weight of dissected individual fat depots and skeletal muscles.

	Control(n = 8)	Manual(n = 9)	Electrical(n = 9)	Control vs. Manual		Control vs. Electrical	Electricalvs. Manual
Body weight, 70 d (g)	252.8±12.9	256.4±15.8	252.3±23.7	0.441	0.847		0.556
Body weight, EP (g)	304.6±5.0	307.0±5.9	295.0±10.2	0.700	0.290		0.270
Fat depots (g)							
Inguinal	1.57±0.11	1.49±0.11	1.27±0.05	0.847	**0.021**		**0.015**
Parametrial	2.98±0.29	2.98±0.43	2.95±0.43	0.773	0.441		0.566
Retroperitoneal	2.33±0.28	2.21±0.24	2.08±0.34	0.773	0.208		0.441
Mesenteric	1.66±0.14	1.72±0.11	1.60±0.17	0.501	0.336		0.310
Fat depots (g/kg body weight)							
Inguinal	5.15±0.35	4.83±0.32	4.33±0.22	1.000	**0.043**		0.058
Parametrial	9.77±0.89	9.64±1.36	9.81±1.20	0.700	0.700		0.895
Retroperitoneal	7.63±0.84	7.15±0.74	6.69±0.46	1.000	0.178		0.310
Mesenteric	5.43±0.39	5.59±0.32	5.38±0.41	0.700	0.923		0.627
Muscles (g)							
EDL	0.14±0.004	0.15±0.004	0.13±0.003	0.923	0.102		**0.047**
Soleus	0.12±0.005	0.13±0.004	0.13±0.003	0.290	0.211		0.627
Tibialis	0.61±0.012	0.63±0.018	0.59±0.019	0.248	0.248		0.093
Muscles (g/kg body weight)							
EDL	0.47±0.011	0.48±0.014	0.46±0.006	0.773	0.336		0.402
Soleus	0.40±0.014	0.42±0.014	0.45±0.013	0.083	**0.007**		0.508
Tibialis	2.02±0.044	2.07±0.07	2.01±0.05	0.923	0.923		0.093

Values are mean ± SEM. EDL, extensor digitorum longus; EP, end point. *P* values were determined with the Mann-Whitney U-test.

### Oral Glucose Tolerance Test

**Figure 1:**
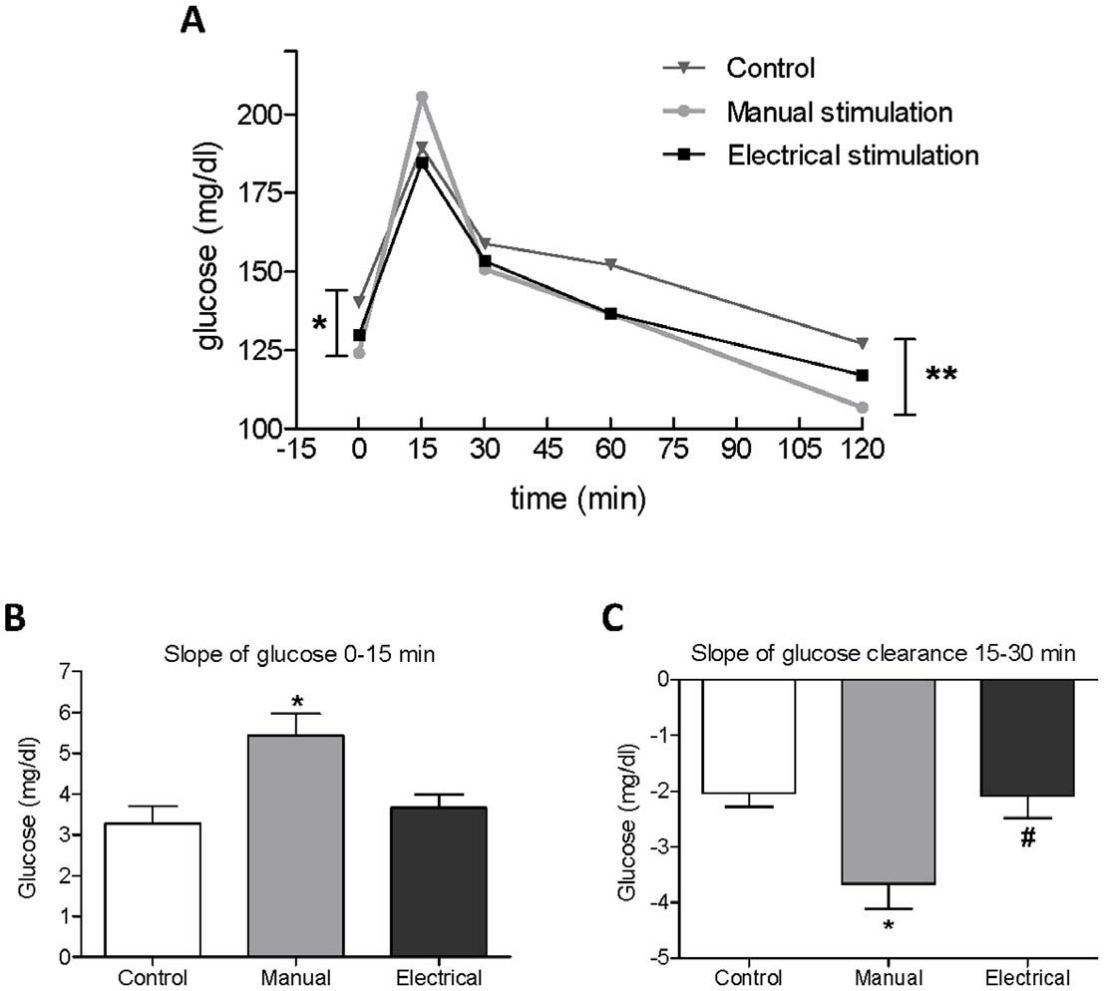
Results of OGTT in rats with DHT-induced PCOS after 4–5 wks of treatment. (A) Glucose concentration in plasma at 0, 15, 30, 60 and 120 min. (B, C) Slope of glucose clearance at 15 min (B) and 30 min (C). Values are mean ± SEM, *P<0.05 manual stimulation vs. controls, **P<0.01 manual stimulation vs. controls. ^#^ *P*<0.05 electrical vs. manual stimulation.

The levels of insulin and pro-insulin and the pro-insulin/insulin ratio did not differ between groups at baseline or during the OGTT (data not shown). However, after 4–5 wks of treatment glucose levels were lower in the manual stimulation group than in controls at time point 0 (124.2±1.9 mg/dl *vs.* 140.4±4.7 mg/dl, *P*  = 0.017) and at 120 minutes (106.8±2.0 mg/dl *vs*. 127.1±3.3 mg/dl, *P*  = 0.001) in the OGTT (Fig. 1A).

There were no differences in the area under curve of glucose or insulin or in the insulin sensitivity index between groups (data not shown). The glucose increase and clearance rate was determined by calculating the slope of glucose concentration from 0–15 and 15–30 minutes of the OGTT. Electrical stimulation did not affect any outcome of the OGTT (Fig. 1B, C). In the manual stimulation group, glucose clearance rate was higher than in controls (Fig. 1B, C).

### Glycogen Content

**Figure 2:**
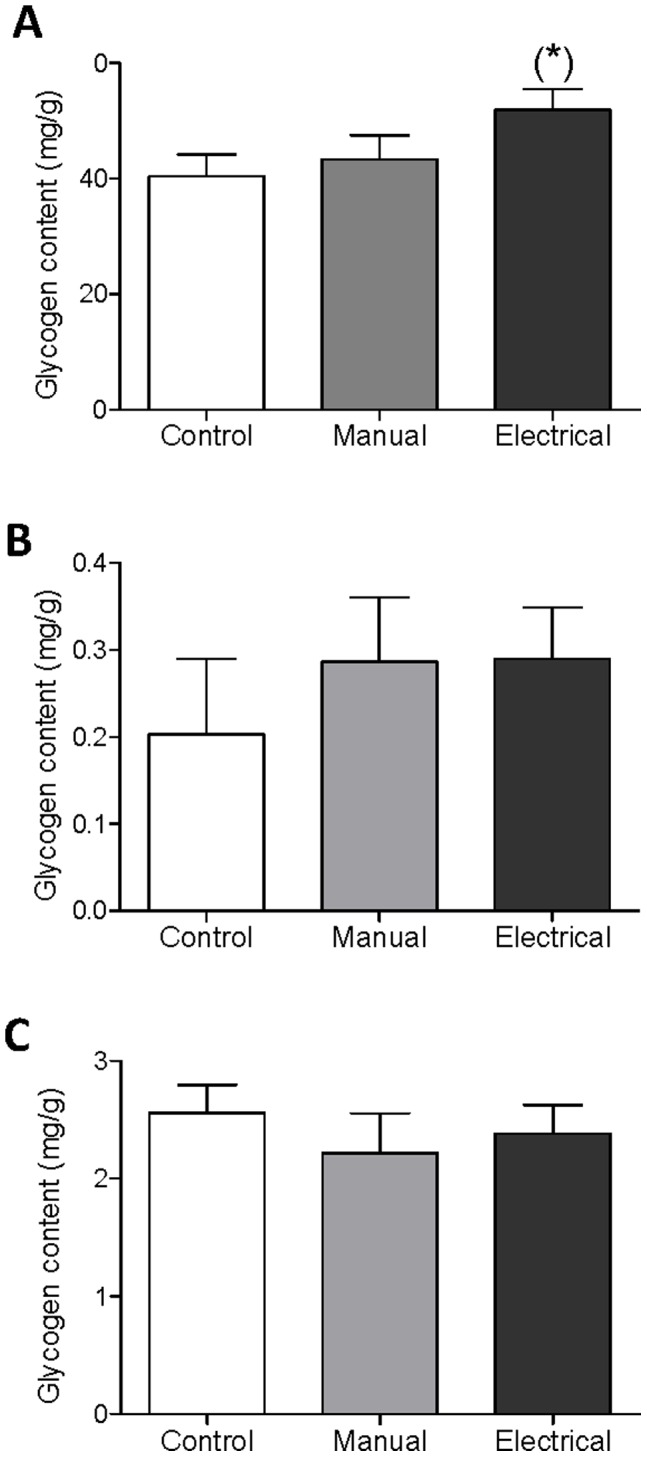
Glycogen content in rats with DHT-induced PCOS after 4–5 wks of treatment. Glycogen content in (A) liver, (B) EDL, and (C) soleus determined with a colorimetric assay. Values are mean ± SEM. (*)*P*  = 0.074 (Mann-Whitney U test).

There were no significant intergroup differences in glycogen content in liver, soleus, or EDL after treatment. However, there was a trend toward a higher glycogen content in liver in the electrical stimulation group than in controls (*P*  = 0.074) (Fig. 2).

### Pancreas and Islets of Langerhans

There were no differences in the mean size of pancreatic islets or the ratio of the insulin-positive area to pancreatic area (control vs. manual stimulation *P*  = 0.386 and *P*  = 0.211, control vs. electrical stimulation *P*  = 0.564 and *P*  = 0.773, manual stimulation vs. electrical stimulation *P*  = 0.122 and *P*  = 0.270, respectively).

### Electrical and Manual Stimulation Differentially Regulate Gene Expression in Soleus and Mesenteric Fat

mRNA expression of 43 target genes related to androgen secretion, glucose transport, MAPK, lipid metabolism, adipokines, and sympathetic/adrenergic activation and five putative endogenous control genes were measured in soleus muscle and mesenteric adipose tissue (Table 1). Expression of nine genes in soleus and five in mesenteric fat were not detected at levels sufficient for statistical analysis (Ct>30).

In soleus muscle, the expression of *Tbc1d1* mRNA was lower in the electrical stimulation group than in controls (Table 3). Although none of the stimulation groups differed from control, mRNA expression of *Mapk1* was lower after electrical stimulation than after manual stimulation. Expression of *Nr4a3* (*P*  = 0.054) tended to be lower in the electrical stimulation group than in controls, although non-significant.

**Table 3:** Relative gene expression in soleus muscle and mesenteric adipose tissue depot.

Gene	Control (n = 8)	Manual(n = 9)	Electrical(n = 9)	Control vs.Manual	Control vs. Electrical	Electrical vs.Manual
Soleus muscle						
*Tbc1d1*	1±0.28	0.67±0.13	0.54±0.06	0.068	0.016	0.354
*Nr4a3*	1±0.23	1.38±0.64	0.4±0.07	0.630	0.054	0.200
*Mapk1/Erk2*	1±0.06	1.18±0.24	0.82±0.05	0.962	0.054	0.031
Mesenteric adipose tissue						
*Nr4a3*	1±0.12	3.36±1.79	1.57±0.27	0.027	0.046	0.923
*Mapk3/Erk1*	1±0.15	2.03±0.32	1.14±0.14	0.006	0.401	0.021
*Adcy3*	1±0.11	1.75±0.3	1.09±0.16	0.012	0.916	0.054
*Gsk3b*	1±0.13	1.6±0.23	1.25±0.14	0.043	0.115	0.312

Presented values are 2^-ΔΔCt^ (mean ± SEM) relative control group. *P* values were determined with the Mann-Whitney U-test. The mean cycle threshold (Ct) value for all genes was 26.84±4.03 (range 8.88–37.45) in soleus muscle and 26.65±3.76 (range 9.81–37.15) in mesenteric adipose tissue.

In mesenteric adipose tissue, the expression of *Nr4a3* mRNA was higher in the electrical stimulation group than in controls, and *Nr4a3, Mapk3, Adcy3*, and *Gsk3b* mRNA was higher in the manual stimulation group than in controls (Table 3).

### Electrical and Manual Stimulation Differentially Regulate the Expression of Proteins in Soleus Muscle and Mesenteric Adipose Tissue

Next, we determined whether changes in insulin sensitivity and gene expression in soleus muscle and mesenteric adipose tissue after electrical and manual stimulation are reflected by alterations in protein expression. Analyses of protein content were limited to GLUT4, AS160, Tbc1d1 and Nr4a3.

**Figure 3:**
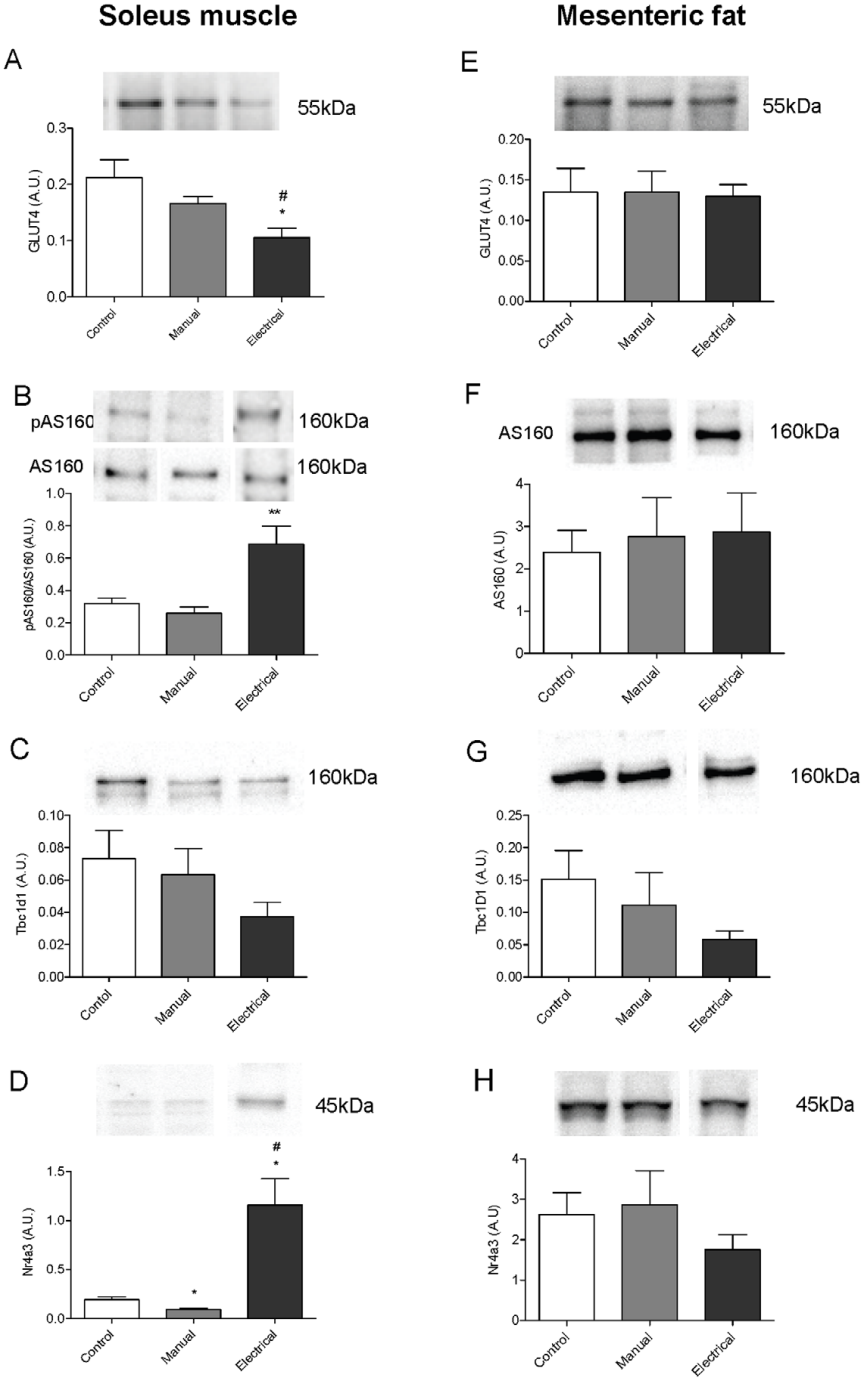
Protein levels in soleus muscle and mesenteric adipose tissue detected by western blot after 4–5 wks of treatment in rats with DHT-induced PCOS. GLUT4 (A, E), pAS160/AS160 ratio (B), AS160 (F), TBC1D1 (C, G), and Nr4a3 (D, H). Representative immunoblots of each protein are shown. Values were normalized to β-actin and are expressed in arbitrary units (A.U.) (mean ± SEM). *P<0.05 vs. controls, **P<0.01 vs. controls, #*P*<0.05 vs. manual stimulation (Mann-Whitney U test).

**Figure 4:**
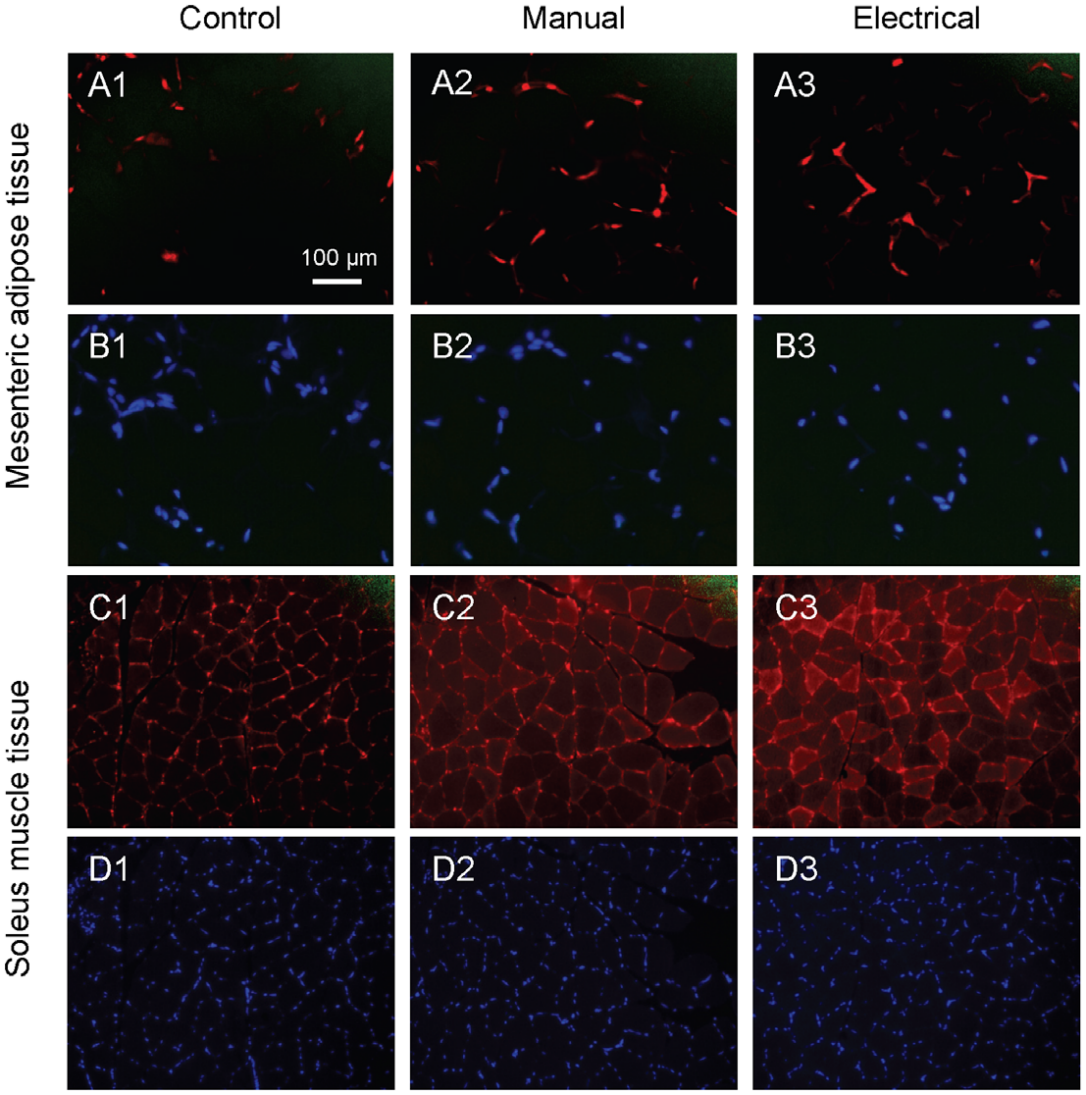
Distribution and expression of GLUT4, determined by immunofluorescence staining, in soleus muscle and mesenteric adipose tissue of rats with DHT-induced PCOS. No visual difference in immunoreactivity is observed in the mesenteric adipose tissue depot (A1–A3). In soleus muscle of control rats, GLUT4 is predominantly localized in the nucleus and cell membrane (C1). Both manual and electrical stimulation increased immunoreactivity in the nucleus, cell membrane, and cytosolic fraction (C2, C3). Staining was notably more intense after electrical stimulation than manual stimulation. Similar results were obtained when the staining was repeated in 3 rats/group for mesenteric adipose tissue and 4 rats/group for soleus muscle. The selected immunofluorescence images are representative of those in randomly selected section from multiple animals. B1–B3 and D1–D3: DAPI staining for nuclei in corresponding rat (A1–A3 and C1–C3). All photographs were taken with a ×20 objective.

In soleus muscle, total GLUT4 content in soleus muscle measured by western blot was significantly lower in the electrical stimulation group than in controls (*P*  = 0.012) or in the manual stimulation group (*P*  = 0.019) (Fig. 3A). However, both electrical and manual stimulation increased GLUT4 expression in soleus muscle (Fig. 4C1–C3), as confirmed by immunofluorescence staining. Both stimulations increased immunoreactivity in the nucleus, cell membrane, and cytosolic fraction (Fig. 4C2, C3), but the staining was notably more intense after electrical stimulation.

The ratio of phosphorylated to nonphosphorylated AS160 in soleus muscle was almost three times higher in the electrical stimulation group than in the manual stimulation group (*P*  = 0.004) and controls (*P*  = 0.005) (Fig. 3B). Nonphosphorylated AS160 in soleus muscle did not differ between groups (Fig. 3B). Protein expression of TBC1D1 in soleus muscle was almost 50% lower in the electrical stimulation group than in controls (*P*  = 0.054) (Fig. 3C). Expression of Nr4a3 in soleus muscle was six times higher in the electrical stimulation group than in the manual stimulation group (*P*  = 0.004) and controls (*P*  = 0.034), while manual stimulation downregulated Nr4a3 (*P*  = 0.012) in soleus muscle compared with controls (Fig. 3D).

In mesenteric adipose tissue, neither electrical nor manual stimulation affected the protein expression (Fig. 3E–H).

## Discussion

The novel finding of this study is that repeated electrical and manual muscle stimulation of acupuncture needles has different effects on insulin sensitivity and signaling mechanisms in an insulin-resistant, obese rat PCOS model. Electrical stimulation decreased the weight of the subcutaneous fat depot, increased the weight of the soleus muscle, and affected the expression of genes and proteins related to insulin signaling pathways in soleus skeletal muscle. In contrast, manual stimulation of needles improved whole-body glucose tolerance and affected gene expression in mesenteric adipose tissue but had no major effect on protein expression.

### Improved Glucose Tolerance Only after Manual Stimulation in DHT-induced PCOS

One of the main characteristics of rats with DHT-induced PCOS is decreased whole-body insulin sensitivity measured by euglycemic hyperinsulinemic clamp [18], [20], [22]. Repeated electrical stimulation of the needles, 3 or 5 times weekly for 4–5 wks, restores insulin sensitivity as measured by the clamp [18], [20]. To avoid influence of an insulin load by the clamp on gene and protein expression, we measured glucose tolerance and insulin secretion (beta-cell function) with an OGTT the week before tissues were collected.

The lack of response on the OGTT in the electrical stimulation group is interesting, as we have repeatedly shown a positive effect on glucose disposal rate measured by the clamp [18], [20]. One may argue that the results of the gold standard clamp method and the OGTT cannot be completely correlated, as the OGTT is less sensitive and provides somewhat different information [31]. In a previous study, we attributed the increased insulin sensitivity after electrical muscle stimulation to an increase in soleus muscle mass, suggesting that muscle twitches evoked have local effects in the muscle [20]. In the present study, we found a similar increase in soleus muscle mass only in the electrical stimulation group. Moreover, only electrical stimulation reduced the weight of the subcutaneous (inguinal) adipose tissue depot. Thus, electrical stimulation has a positive effect on body composition that may affect insulin sensitivity although not reflected by the OGTT in this study, although it was in previous euglycemic clamp experiments. Other potential explanations for the differences in response after electrical stimulation in the OGTT and the clamp include small sample sizes or that the effect of treatment involves increased responsiveness to high insulin levels rather than sensitivity.

Although the area under the curve did not differ between the groups, the glucose concentration at 0 min (fasting) and at 120 min of the OGTT was lower in the manual stimulation group than in controls with no differences in concurrent insulin levels. This could represent improved hepatic insulin sensitivity or decreased pancreatic glucagon release with lowering of high endogenous fasting glucose production, as in type 2 diabetes [32]. Because the sample volume was limited, we could not measure circulating glucagon. The increased glucose clearance rate after manual stimulation, together with lower glucose level at 2 h indicates improved glucose tolerance and possibly increased peripheral insulin sensitivity after manual stimulation, with no effect on pancreatic insulin function.

Next we aimed to elucidate the molecular mechanisms of action of the improved insulin sensitivity after 4–5 wks of manual stimulation in this study and after electrical stimulation in previous studies [18], [20], [22]. Muscle contractions during electrical stimulation of needles may stimulate glucose uptake by an insulin-independent pathway, as occurs after exercise [16], [18], [20], [33], [34]. Whether manual stimulation also activates insulin-independent pathways in skeletal muscle or adipose tissue has not previously been investigated.

First, we performed a more extensive screening by analyzing the expression of genes related to androgen secretion, glucose transport, MAPK, lipid metabolism, and sympathetic/adrenergic activation in soleus skeletal muscle and mesenteric adipose tissue (Table 1). Since the assessed gene expression revealed changes in several genes associated with insulin signaling, we analyzed expression of GLUT4 and its most proximal effectors (AS160, TBC1D1, and Nr4a3) by western blot. This was based on the changes in gene expression of Tbc1d1 and Nr4a3 with an addition of AS160, the homologue of Tbc1d1.

### Molecular Effects of Electrical and Manual Stimulation in Soleus Skeletal Muscle

Immunofluorescence staining of GLUT4 in soleus muscle was increased after electrical stimulation and, to a lesser extent, after manual stimulation. These results are consistent with our previous finding that electrical muscle stimulation increases GLUT4 protein expression after insulin stimulation (clamp) [18]. Staining was stronger in cytosolic compartments, cell membranes, and nucleus after both stimulation techniques than in controls, which at least in part reflect increased protein expression as well as translocation. However, in contrast to our previous findings, neither gene nor protein expression of GLUT4/Slc2a4 aligned with immunofluorescense staining, indicating a stronger effect on translocation than expression. Data from membrane fractionation experiments are needed to elucidate this effect. One plausible explanation for the discrepancy in GLUT4 protein expression in soleus muscle in the present and previous study may be the absence of high insulin levels during the clamp, driving GLUT4 expression and translocation. The inconsistency between western blot and immunofluorescence might therefore reflect differences in experimental conditions or the fact that more GLUT4 in the electrical stimulation group is membrane bound as a consequence of translocation.

Two of the most distal proteins in the insulin signaling cascade closely linked to GLUT4 are AS160 and the related homologue TBC1D1, both downstream of Akt (protein kinase B). Non-phosphorylated AS160 functions as a brake on GLUT4 translocation. Phosphorylation in response to insulin, AMP-activated protein kinase (AMPK) activator 5-aminoimidazole-4-carboxamide-1-β-_D_-ribofluranotide (AICAR), or exercise-associated contraction inactivates this brake to allow glucose transport [35], [36], [37]. In support of this hypothesis, electrical muscle stimulation increased the ratio of pAS160/AS160 in the soleus muscle, indicating reduced functional activity, increased GLUT4 translocation, and possibly increased insulin sensitivity. Unphosphorylated TBC1D1 may have a role in GLUT4 traffic [36], and reduced expression of TBC1D1 in skeletal muscle increases glucose uptake and oxidation of fatty acids [38]. TBC1D1 protein expression is several times more abundant in skeletal muscles than in fat and, like AS160, insulin, muscle contraction, and AICAR increase phosphorylation (inactivation of brake) in vivo [39]. Here, electrical stimulation significantly lowered expression of *Tbc1d1* mRNA in soleus muscle, and manual stimulation tended to lower it. The reduced *Tbc1d1*mRNA expression in soleus muscle is supported by the reduction in protein expression (*P*  = 0.054). The lack of change in *Slc2a4* (GLUT4) mRNA expression suggests a potential role for TBC1D1 in the posttranscriptional modifications of GLUT4 translocation. Consistent with our hypothesis, these results also indicate that low-frequency electrical stimulation causing muscle contractions improves glucose uptake and oxidation of fatty acids in muscle.

Lastly, NR4A3, a member of the NR4A family of orphan nuclear receptors, is widely expressed in different cell types and mediates diverse biological processes [40], [41]. *Nr4a3* expression is reduced in skeletal muscle and adipose tissue in multiple rodent models of insulin resistance, while increased expression of *Nr4a3* increases insulin responsiveness and GLUT4 translocation [41]. At the protein level, electrical but not manual stimulation dramatically increased Nr4a3 expression in soleus muscle. This finding is consistent with previous studies of electrical stimulation causing muscle contractions and exercise and might indicate increased insulin sensitivity [42], [43].

### Molecular Effects of Electrical and Manual Stimulation in Mesenteric Adipose Tissue

Although the molecular effects of electrical and manual stimulation were less pronounced in the mesenteric adipose tissue than in soleus muscle, manual stimulation had the strongest effect on gene expression in mesenteric adipose tissue. mRNA expression of *Adcy3* and *Erk1* increased in adipose tissue by manual stimulation, indicating that the effects after manual acupuncture stimulation may involve modulation of autonomic activity and MAPK signaling. Also, expression of *Nr4a3* mRNA in mesenteric adipose tissue was increased by both manual and electrical stimulation, indicating increased insulin responsiveness and GLUT4 translocation by both methods [41]. However, protein expression was not changed in mesenteric adipose tissue.

### Acupuncture Mechanism

Both manual and electrical stimulation cause afferent activity in Aα, β, δ, and unmyelinated C-fibers [44], [45], similar to some of the effects of exercise [46]. Both stimulations may, via supraspinal pathways, directly or indirectly modulate the sympathetic output to target organs [47], [48], [49]. Electrical stimulation of the needles enhances insulin sensitivity in rats, probably through mechanisms related to activation of afferent sensory nerve fibers and modulation of efferent sympathetic nerve activity [19], [21], [50]. In addition, acupuncture with combined electrical and manual stimulation decreases high sympathetic nerve activity in women with PCOS [51]. This effect may be related, at least in part, to the release of β-endorphin [52], [53].

The differential effects of manual and electrical acupuncture stimulation in this study could reflect differences in stimulation duration, given that needles were manually stimulated every 5 min by compared with continuous low-frequency electrical stimulation. For further understanding, the acute effects of the two stimulation modalities with similar duration should be explored. It is also necessary to evaluate the experimental findings in a clinical setting. Although acupuncture would never be a complete alternative to exercise as a first-line therapy, some patients cannot exercise vigorously enough to improve their insulin sensitivity. Also, we have reason to believe that acupuncture enhances the beneficial effects of exercise. Thus, the combination of these treatments may be optimal [54].

### Conclusion

Electrical and manual muscle stimulation affect glucose homeostasis through different mechanisms in rats with DHT-induced PCOS. Repeated electrical stimulation of acupuncture needles regulated key functional molecular pathways related to insulin sensitivity in soleus muscle and mesenteric adipose tissue to a larger extent than manual stimulation. Manual stimulation improved whole-body glucose tolerance as measured by OGTT, an effect that was not observed after electrical stimulation, but did not affect molecular signaling pathways to the same extent as electrical stimulation. Although more functional signaling pathways related to insulin sensitivity were affected by electrical stimulation, our findings suggest that manual stimulation of acupuncture needles has a greater effect on glucose tolerance. The underlying mechanism of the differential effects of the intermittent manual and the continuous electrical stimulation remains to be elucidated.
